# Supplementary material for: Characterization of Clostridioides difficile Persister Cells and Their Role in Antibiotic Tolerance
Source: Microorganisms. 2024 Jul 9;12(7):1394. doi: 10.3390/microorganisms12071394 (PMC11279270; doi:10.3390/microorganisms12071394)
Supplement: Supplementary file 1 [file microorganisms-12-01394-s001.zip › microorganisms-3084564-supplementary.pdf]

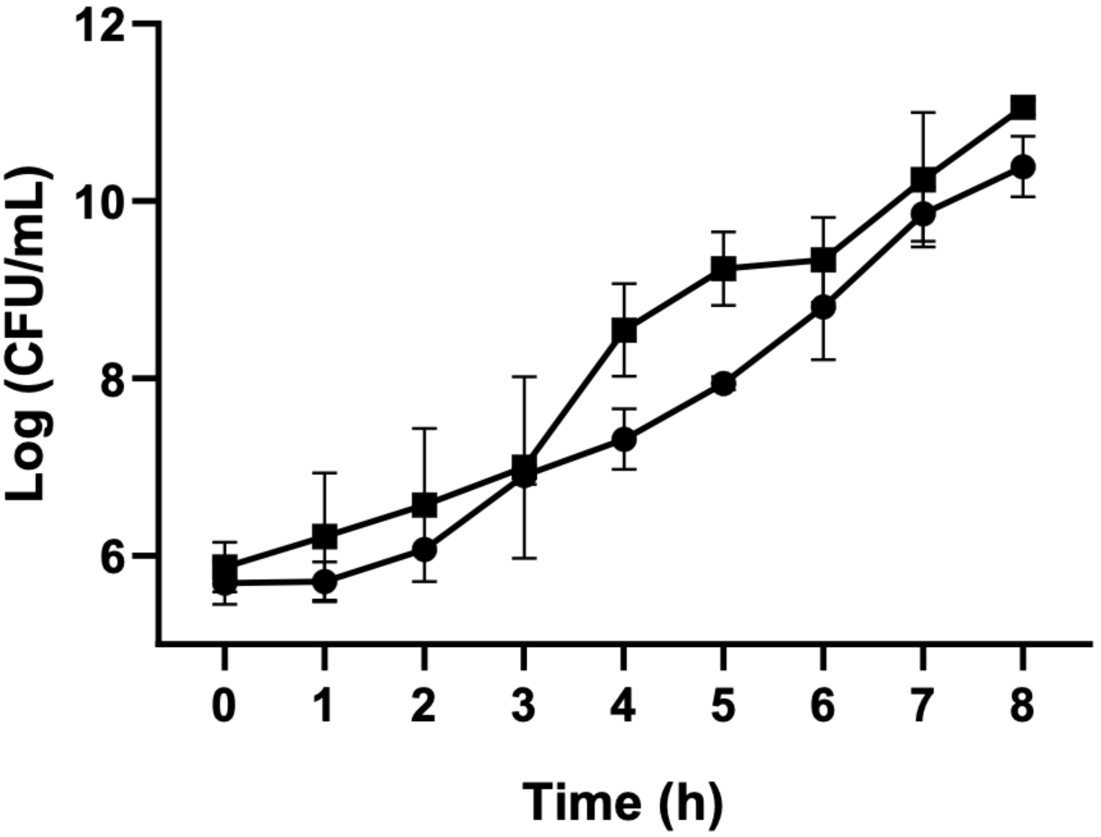

Figure S1. *C. difficile* strains growth curves. Growth curves were made for the wild-type and Δ*spo0A* strains. First, BHIS broths were inoculated with overnight cultures of each strain (inoculum size = 1% v/v), then an aliquot was taken, serially diluted, and seeded on BHIS plates at 0, 1, 2, 3, 4, 5, 6, 7 and 8 h of incubation. After 16 h, colonies in plates were counted, and CFU/mL was calculated for each time point and strain. (n=3).

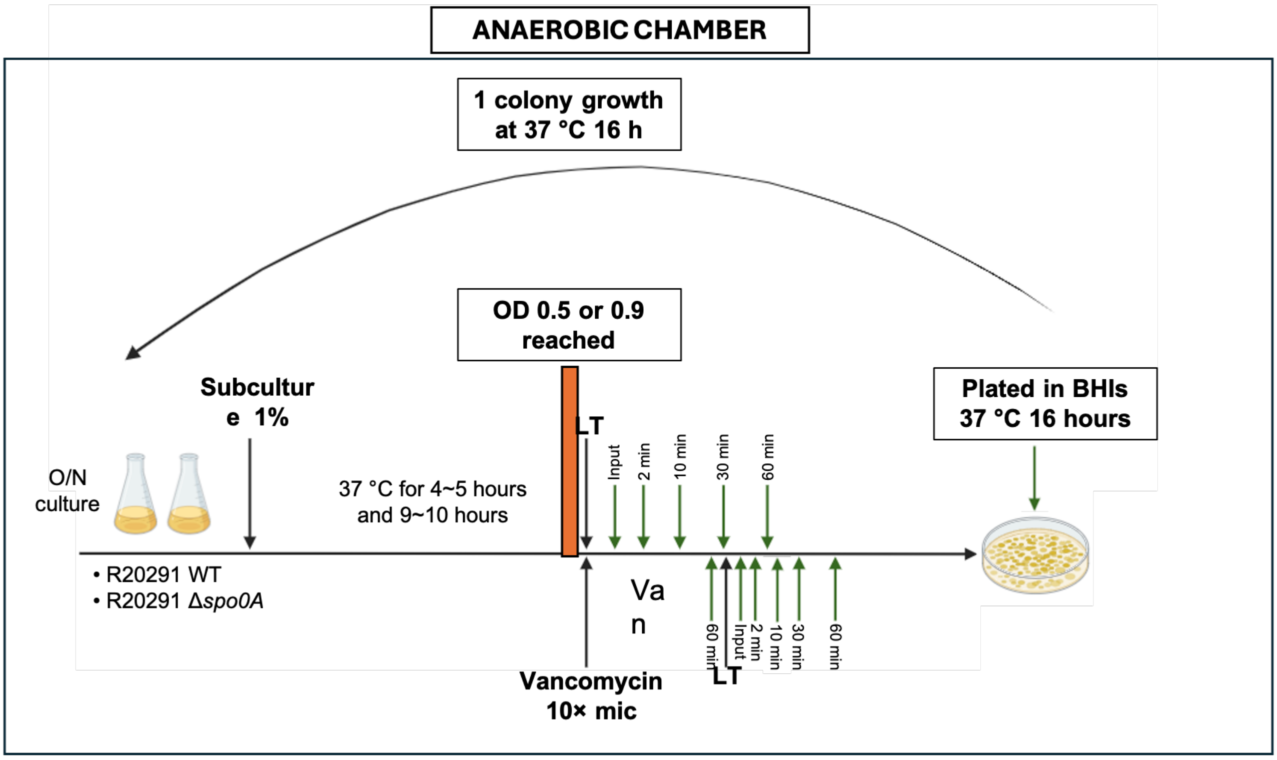

Figure S2. Schematic timeline methods.

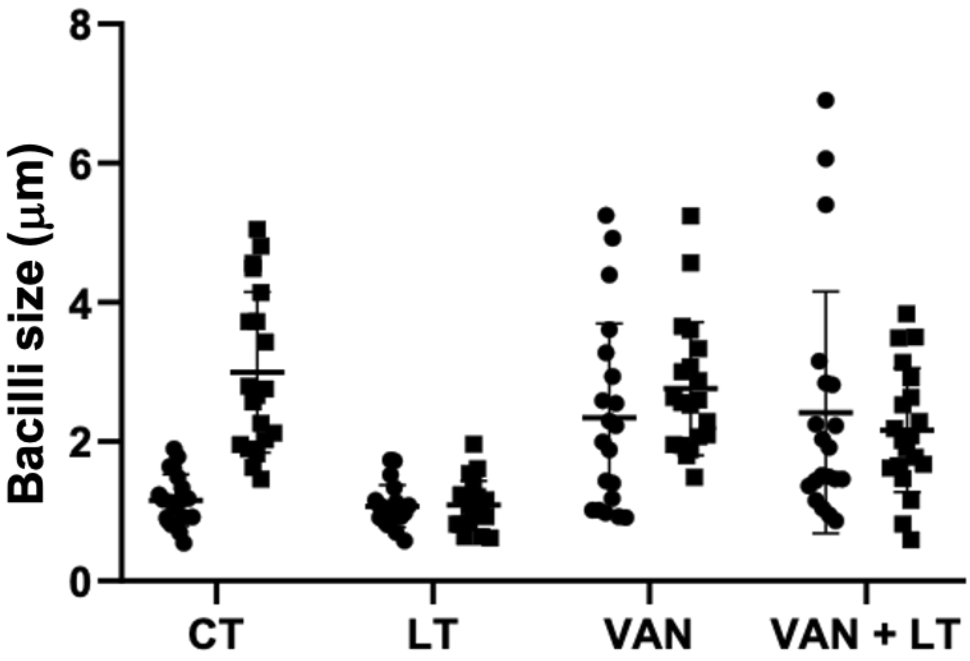

Figure S3. Bacteria count using ImageJ with the scale tool. A total of 20 bacilli per sample were counted for plotting in exponential (black circle) or stationary (black squares) phase. CT: control growth, LT: lysis treatment during each growth phase, VAN: treatment with the antibiotic Vancomycin alone in each growth phase, VAN + LT: Vancomycin treatment for 60 minutes followed by enrichment with lysis treatment. representative data are shown.

Table S1. Primer sequences used for qPCR analysis.

Primer	Sequence
qPCR_clpB_F	CCCAGAAGGCACATATGATGCA
qPCR_clpB_R	CCTACACCTGGCTCACCAATCA
qPCR_clpC_F	TTACACGGGCAACCAGAATCAA
qPCR_clpC_R	TGGCTGTTTTCCCTACACCTGG
qPCR_clpX_F	TGCAATGGCAGATGCTACATCA
qPCR_clpX_R	TGGGTTTTCAGATTTTCTTGCA
qPCR_clpP1_F	TATGGGAGCATTCTTGTTGGCA
qPCR_clpP1_R	AATGGCTGACCAGTTCTTTCAG
qPCR_clpP2_F	CCCTGGTGGCTCTGCTACATCA
qPCR_clpP2_R	CCTCCCATTGGCTGATGAATCA
qPCR_LON_F	ATGAAGGAATCTGCGAAGACAGG
qPCR_LON_R	TGCTGAGATTACTGCAAGAGCCA
qPCR_mazF_F	CGCTGATTTAAGTCCAGTTGTTGG
qPCR_mazF_R	GCCCATATTCATTGGAACTTATCT
qPCR_relE_F	TACAAACGAGCCATCAAGCGTG
qPCR_relE_R	GTCCGGGAGAATGTGACATTCC
qPCR_COG_F	TGGACATCATTTCACTAGCCTTGG
qPCR_COG_R	AAACGCAAGTGATGCCAGAATTAA
qPCR_FIC_F	TTCCCTACGAATCTACCTCATGGAG
qPCR_FIC_R	TGTCCACCTACTGTTATACCATCAA
